# The Use of an Abdominal Bandage in the Second Stage of Labor

**Published:** 1887-01

**Authors:** 


### *THE USE OF AN ABDOMINAL BANDAGE IN THE SECOND STAGE OF LABOR.*

Up to the commencement of the second stage of labor, the uterus alone is concerned in dilating the neck, but it then seems to call in aid the contraction of the abdominal muscles, and consequently both the pain and the bearing down are carried to a much higher degree. The pains are stronger, yet nevertheless the woman assists them by voluntarily contracting the abdominal muscles, and as the pains grow stronger and the pains seem to be tedious, then the woman will often call on her physician for help. I believe that this assistance can be rendered by the use of an abdominal bandage, and that by it, in the second stage of labor, we may not only lessen the suffering of our patient, but, at the same time, shorten the duration of labor. Consequently, I use the abdominal bandage for a twofold purpose: First, to lessen the suffering of my patient. To accomplish this, I apply it at or before the commencement of the second stage of labor, making it just tight enough to be comfortable to my patient. Second, to shorten the duration of labor. To accomplish this end, I tighten the bandage when the abdominal muscles are called upon to assist the uterus in expelling its contents. In my first cases, I used simply an ordinary linen towel, which I put around the abdomen of the woman and secured with pins, which I unpinned and tightened as the case demanded. I now use a bandage which I constructed for that purpose, which resembles in shape the lower half of a corset, except I have it open on the side, making a back and abdominal piece, which I unite by means of straps and buckles. Having it open on both sides, I can adjust it more easily to fit different-sized patients.—*Welker, in Therapeutic Gazette.*

Following, Dr. Welker reports two cases in which he used the abdominal bandage above-mentioned with astonishing results. The results are: increased frequency and strength of pains in cases where pains had been slow and feeble, with consequent rapid delivery. (Theoretically, the principle of giving support to the abdominal muscles and the uterus during the expulsive stage can but add to the strength and efficiency of expulsive efforts.)

